# Computer-Aided Detection for Chest Radiography to Improve the Quality of Tuberculosis Diagnosis in Vietnam’s District Health Facilities: An Implementation Study

**DOI:** 10.3390/tropicalmed8110488

**Published:** 2023-10-29

**Authors:** Anh L. Innes, Andres Martinez, Xiaoming Gao, Nhi Dinh, Gia Linh Hoang, Thi Bich Phuong Nguyen, Viet Hien Vu, Tuan Ho Thanh Luu, Thi Thu Trang Le, Victoria Lebrun, Van Chinh Trieu, Nghi Do Bao Tran, Zhi Zhen Qin, Huy Minh Pham, Van Luong Dinh, Binh Hoa Nguyen, Thi Thanh Huyen Truong, Van Cu Nguyen, Viet Nhung Nguyen, Thu Hien Mai

**Affiliations:** 1 FHI 360 Asia Pacific Regional Office, Bangkok 10330, Thailand; 2 FHI 360, Durham, NC 27701, USA; amartinez@fhi360.org (A.M.); xgao@fhi360.org (X.G.); ndinh@fhi360.org (N.D.); 3 FHI 360 Vietnam, Hanoi 10000, Vietnam; hlinh@fhi360.org (G.L.H.); nphuong@fhi360.org (T.B.P.N.); vhien@fhi360.org (V.H.V.); ltuan@fhi360.org (T.H.T.L.); ltrang@fhi360.org (T.T.T.L.); vlebrun@fhi360.org (V.L.); tchinh@fhi360.org (V.C.T.); tnghi@fhi360.org (N.D.B.T.); thuhien@fhi360.org (T.H.M.); 4 Stop TB Partnership, Grand-Saconnex, 1218 Geneva, Switzerland; zhizhenq@stoptb.org; 5 United States Agency for International Development/Vietnam, Hanoi 10000, Vietnam; mpham@usaid.gov; 6 Vietnam National Lung Hospital, Hanoi 10000, Vietnam; dinhvanluong66@gmail.com (V.L.D.); nguyenbinhhoatb@yahoo.com (B.H.N.); thanhhuyenntp@gmail.com (T.T.H.T.); cu_nguyen_van@yahoo.com (V.C.N.); vietnhung@yahoo.com (V.N.N.); 7 Pulmonology Department, University of Medicine and Pharmacy, Vietnam National University, Hanoi 10000, Vietnam

**Keywords:** tuberculosis, artificial intelligence, radiography, calibration, innovative diagnosis

## Abstract

In Vietnam, chest radiography (CXR) is used to refer people for GeneXpert (Xpert) testing to diagnose tuberculosis (TB), demonstrating high yield for TB but a wide range of CXR abnormality rates. In a multi-center implementation study, computer-aided detection (CAD) was integrated into facility-based TB case finding to standardize CXR interpretation. CAD integration was guided by a programmatic framework developed for routine implementation. From April through December 2022, 24,945 CXRs from TB-vulnerable populations presenting to district health facilities were evaluated. Physicians interpreted all CXRs in parallel with CAD (qXR 3.0) software, for which the selected TB threshold score was ≥0.60. At three months, there was 47.3% concordance between physician and CAD TB-presumptive CXR results, 7.8% of individuals who received CXRs were referred for Xpert testing, and 858 people diagnosed with Xpert-confirmed TB per 100,000 CXRs. This increased at nine months to 76.1% concordant physician and CAD TB-presumptive CXRs, 9.6% referred for Xpert testing, and 2112 people with Xpert-confirmed TB per 100,000 CXRs. Our programmatic CAD-CXR framework effectively supported physicians in district facilities to improve the quality of referral for diagnostic testing and increase TB detection yield. Concordance between physician and CAD CXR results improved with training and was important to optimize Xpert testing.

## 1. Introduction

Tuberculosis (TB) is one of the top 10 causes of death worldwide, and it was the leading cause of death—until COVID-19—due to a single infectious agent [1]. Approximately 10.6 million people fell ill with TB in 2021, but only 6.4 million were newly diagnosed with and notified as having TB at the global level [1]. Improving diagnosis is an urgent priority to address one of the biggest barriers to ending TB.

In recent years, the increased array of TB diagnostic methods and emphasis on early detection have moved chest radiography (CXR) to the front of diagnostic algorithms [2]. CXR abnormalities can guide confirmatory diagnostic testing for TB, but the accuracy of this decision depends on the quality of interpretation. Using computer-aided detection (CAD) artificial intelligence (AI) to interpret CXR films has the potential to standardize the quality of TB case finding. Early versions of CAD software were not as accurate as expert human readers [3,4], but the best software products now perform at or above the accuracy of human experts [5,6]. In March 2021, the World Health Organization (WHO) conditionally recommended CAD in place of human readers to interpret digital CXRs for TB among individuals 15 years or older [7]. The guideline defines two use cases for CAD: asymptomatic populations (screening) and symptomatic populations with TB risks and symptoms (triage). Due to variable performance in different settings, implementers are advised to calibrate CAD threshold scores to optimize yield for TB detection and ensure accurate referral for diagnostic testing [8]. CAD for CXR interpretation is rapidly expanding, with new and updated software each year [9] and increasing numbers of research studies conducted in high TB-burden settings [10,11]. Recent studies highlight the importance of threshold calibration, showing variable CAD diagnostic performance with different versions of software and population subgroups [12,13].

WHO describes four models for integrating CAD to screen or triage for confirmatory TB diagnosis [14]: “(1) CAD for initial screening, with any abnormal result referred to a human reader for final interpretation; (2) CAD for initial screening, with a portion of all results verified by a human reader (e.g., all abnormal CXRs and 10% of normal CAD results); (3) CAD replacing a human reader, with all abnormal results referred for diagnosis; and (4) CAD and human reading in parallel, with an abnormal result from either reading referred for diagnosis”. In radiology, AI ideally complements human expertise [15], with a broad range of potential applications that includes improving diagnostic accuracy [16] and augmenting radiology training [17]. Frameworks have been proposed to develop sustainable AI-driven solutions in lower-resource settings [18]. AI integration of image and clinical data can produce a more holistic patient assessment, improving clinical care [19]. CAD for CXR interpretation in high TB-burden, low-resource settings can support clinical decisions by simplifying results (TB-presumptive or not TB) to indicate confirmatory diagnostic testing. The value of CAD support differs depending upon the user, with higher benefit for those with less experience, such as non-radiology physicians [20].

In Vietnam, there is a large gap between the estimated TB incidence (169,000 in 2021) and the number of people notified with TB each year—approximately 40% pre-COVID-19 and worsening to 54% in 2021, during the nadir of the pandemic [21]. To increase TB diagnosis, the Vietnam National Tuberculosis Program (NTP) developed the “Double X” (2X) strategy that uses CXR to indicate GeneXpert (Xpert) (Cepheid, Sunnyvale, CA, USA) testing for pulmonary TB disease. Xpert is a commonly used, WHO-recommended rapid diagnostic (WRD) that is sensitive and specific for diagnosing TB disease [22]. The 2X algorithm leverages the high sensitivity of CXRs to detect TB abnormalities combined with highly specific Xpert diagnosis, for high-yield TB detection [23].

**Figure 1:**
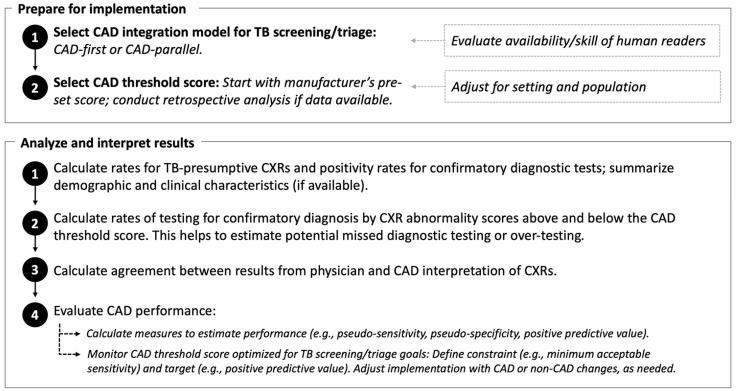
Framework for programmatic computer-aided detection (CAD) artificial intelligence to interpret chest radiography (CXR) for tuberculosis (TB) abnormalities.

The Vietnam NTP piloted the 2X strategy in research studies and found that it was high yield for Xpert-confirmed TB [24,25]. However, the rate of “TB-presumptive” CXRs during programmatic implementation varied widely across provinces, with a range of yields for TB detection. Although TB prevalence differs for Vietnam’s three regions [26,27], the variability in 2X results flagged possible issues with the quality of CXR interpretation. To address this, CAD was first applied retrospectively to interpret CXRs from 2X community campaigns in 2020 and analyzed for agreement with national and provincial radiologists. CAD was then integrated into the real-time workflow of 2X community campaigns in 2021–2022 to support physicians with CXR interpretation. From these results and experience, a CAD framework was developed for routine programmatic implementation (Figure 1). From April through December 2022, the framework was applied in an implementation study for facility-based 2X case finding to determine if CAD integration improved the quality of physicians’ CXR interpretation; the rate of Xpert referral; and the yield for Xpert-confirmed TB disease. Agreement between physician and CAD CXR results was calculated to examine physician uptake of CAD support.

## 2. Materials and Methods

### 2.1. Study Design 

This multi-center implementation study integrated CAD for CXR interpretation into facility-based TB case finding in Vietnam from April through December 2022. The study was part of a larger project implementing the 2X strategy in communities and facilities that started in March 2020, funded by the United States Agency for International Development.

### 2.2. Setting

Five provinces were selected for their representation of the country’s southern (An Giang, Dong Thap, Tay Ninh, Tien Giang) and northern (Thai Binh) regions; baseline provincial TB notification rates comprised approximately 13% of notified TB in Vietnam. District facilities were selected based upon the availability of digital chest radiography, patient volume (ranging from 1000–2280 CXRs per facility each month) and the facility’s commitment to integrate CAD into routine TB case finding. Five, seven, and eight district facilities launched CAD integration at three, six, and nine months of implementation, respectively; the expansion to additional facilities over time was aligned to the capacity for project monitoring and support.

### 2.3. Facility 2X Screening and Triage Algorithms

The CAD-integrated 2X strategy applied CXR and Xpert testing for a range of vulnerable populations six years and older in the facility setting. The first step was the evaluation of clinical risks for TB and respiratory symptoms and, if indicated, CXR; those with TB-presumptive CXRs were referred for Xpert testing. Vulnerable populations evaluated by CAD-integrated 2X comprised the following: (1) newly diagnosed diabetes outpatients or diabetics with poorly controlled glucose (elevated hemoglobin A1C or random blood glucose) were screened for TB symptoms; (2) inpatients with lung disease (no symptoms required) and outpatients in general medical clinics with respiratory symptoms (of any duration) were evaluated by 2X; (3) elderly individuals ≥60 years old, smokers (≥10 cigarettes per day), and those with alcohol use disorders (≥six servings at a time, each week for the preceding three months) underwent 2X evaluation if they had TB symptoms or no CXR within six months. (4) CAD analysis was also applied to CXRs for individuals without TB risks or symptoms, with the rationale to reach vulnerable populations who may have missed the initial screening by symptoms and clinical risk factors.

### 2.4. CXR Interpretation

CXRs were obtained by a variety of radiography machines, which differed by facility, and interpreted by an on-site, district-level physician who was a radiologist or a clinician (diabetes, general, or TB physician). CXR images were interpreted by physicians as “not TB” or “TB-presumptive”; digital images were uploaded as uncompressed Digital Imaging and Communications in Medicine (DICOM) files to a password-protected server.

### 2.5. CAD Analysis

DICOM CXR images were analyzed offline using qXR [28] version 3.0 (Qure.ai, Mumbai, India). qBoxes were installed on stationary radiology picture archiving and communication systems (PACS). qXR analysis resulted in an output “abnormality score” between 0.00 and 1.00 that increased with the level of CXR abnormality. The manufacturer’s pre-set threshold score for TB (0.50) produced a binary outcome for which qXR ≥ 0.50 was “TB-presumptive” and qXR < 0.50 was “TB negative”. Using the programmatic framework that was developed from retrospective and real-time CAD implementation in 2020–2022, the CAD integration model was selected for 2X facility case finding, with WHO models as reference [7]. To accommodate the facility workflow, the CAD-parallel model was selected in which CAD software and physicians both interpreted all CXRs; it was not mandatory to incorporate the CAD result into the final decision for Xpert testing, which was made by the on-site physician. The decision to obtain an CXR in the facility setting, its interpretation, and referral for Xpert testing were made by different people in separate locations; this workflow was better aligned with the parallel, rather than sequential, CAD model. A qXR ≥ 0.60 threshold score was selected for CAD-parallel implementation.

### 2.6. Xpert Testing

Xpert testing was ordered by an on-site physician after reviewing the CXR and clinical presentation. Participants referred for Xpert testing (by CXR or symptoms) produced a single-spot specimen that was analyzed on site or transported to a nearby facility with Xpert capacity. Xpert MTB/RIF and Xpert Ultra cartridges were provided by the NTP. Trace positive samples were handled according to the national guideline, in which Xpert testing was conducted on a second sputum specimen, followed by provincial committee evaluation of the individual’s clinical presentation, CXR interpretation, and Xpert results.

### 2.7. Data Sources

CAD results and DICOM files were downloaded from each qBox onto a password-protected external hard drive during implementation and then uploaded onto a password-protected server (Microsoft SharePoint, https://www.microsoft.com/en-us/microsoft-365/sharepoint/collaboration, accessed on 1 January 2023). All data analyzed for this implementation study were de-identified. Very limited demographic data were collected on site during the routine, programmatic implementation; these data were not available for analysis.

### 2.8. Data Analysis

Data were analyzed using STATA 17 [29] and R 4.3.0 [30]. Rates for TB-presumptive CXRs and Xpert positivity were calculated for physician and CAD CXR results. The rate of Xpert testing was calculated for CXRs with abnormality scores above and below the qXR TB threshold, which reflected physicians’ referral decisions for Xpert testing. Agreement was measured between physician and CAD CXR results using Cohen’s kappa. The test of proportions was used to compare CXR TB-presumptive rates, Xpert testing rates, and agreement by physician and CAD CXR results. Data were analyzed at quarterly intervals to enable timely monitoring for performance improvement, since this was the first programmatic implementation of CAD for CXR interpretation in these facilities.

For CAD performance measures, analyses were limited to observations with a positive or negative Xpert result (excluding trace) and a valid qXR abnormality score, which predicted the dichotomous Xpert outcome. Individuals with trace positive results were excluded from analysis given the potential complexity of interpretation in a high TB-burden setting. Using Xpert as the bacteriological reference, we calculated sensitivity, specificity, positive predictive value (PPV), and accuracy (number of true positives plus true negatives among all positives plus all negatives) at each threshold score. Areas under the receiver operating characteristics curve and precision recall curve (AUROC, PRAUC) were also calculated [31,32]. Confidence intervals were based on 2000 bootstrap replicates. Simulations conducted by Google suggest that Iterative Threshold Score Calibration (ITSC) is an alternative to conducting prospective clinical trials to identify optimal thresholds [33]. We adapted this approach to monitor implementation, using the ITSC methodology of setting a constraint (e.g., sensitivity) and target (e.g., PPV). An optimal threshold at which the highest PPV could be reached, closest to the target of 20% PPV, was selected if the sensitivity of detecting an Xpert-positive result was greater than 95%. The optimal threshold prioritized user-defined measures (sensitivity and PPV) to monitor implementation. Changes in the optimal threshold may indicate changes in the population screened and/or changes in human decisions for CXR interpretations and Xpert referral. Please see Supplementary Materials for the computer code developed for this manuscript.

## 3. Results

### 3.1. Results Overall for April–December 2022

**Figure 2:**
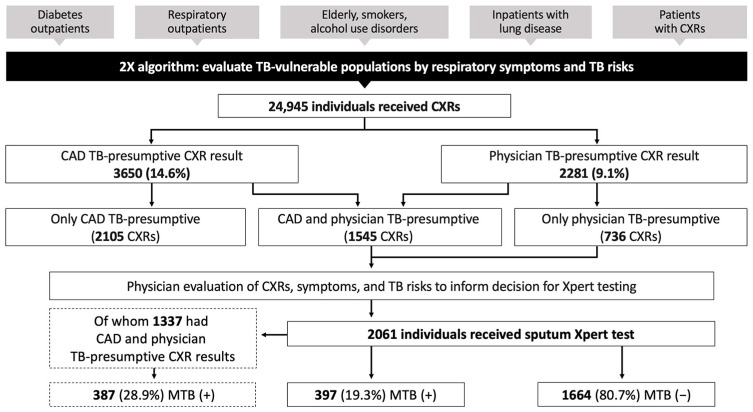
CAD integration into the Double X algorithm in district facilities. Data are from nine months (April–December 2022) of CAD-parallel implementation, in which both CAD and physicians interpreted all CXRs for TB vulnerable populations. 2X = Double X; MTB = mycobacterium tuberculosis; Xpert = GeneXpert; (+) = positive by Xpert test; (−) = negative by Xpert test.

Using CAD-parallel integration to support 2X facility case finding, we found that Xpert testing was conducted for CXRs read as TB-presumptive by physicians alone or TB-presumptive by physicians and CAD (Figure 2). The 2105 CXRs read as TB-presumptive by CAD alone were not referred for Xpert testing. Physicians interpreted 9.1% CXRs as TB-presumptive, which was lower than the 14.6% CXRs interpreted as TB-presumptive by CAD. The overall Xpert positivity rate was 19.3%, which was lower than the 28.9% Xpert positivity for the subset with CAD and physician TB-presumptive CXRs.

### 3.2. CXR Abnormality and Xpert Positivity Analyzed by CAD and Physician CXR Interpretation

Data analysis by quarter showed that rates of CAD TB-presumptive CXRs were significantly higher than physician TB-presumptive CXRs, and both rates were highest at nine months (Table 1). Xpert positivity rates for facility case finding increased after the first three months of CAD integration (from 11.0% to 21.1% and 22.0%). Across all quarters, CXRs with concordant CAD and physician TB-presumptive results had higher Xpert positivity rates than Xpert positivity overall. In contrast, CXRs with physician TB presumptive but CAD non-TB results had much lower Xpert positivity rates.

### 3.3. Analysis of Xpert Testing by CXR Abnormality Scores Relative to the CAD TB Threshold

The Xpert testing rates were 7.8%, 7.3%, and 9.6% across nine months of implementation (Table 2). Despite the slight increase in Xpert testing rates overall, the proportion of individuals who did not receive Xpert testing but had CXR abnormality scores higher than the CAD TB threshold remained high and was 56.6% at nine months. Among those with Xpert tests, the proportion with CXR abnormality scores < 0.60 was 54.7% after three months, which decreased but was still high at six (32.9%) and nine (29.6%) months.

### 3.4. Analysis of Agreement between Physician and CAD CXR Results

Agreement between physician and CAD CXR results increased from three to six and nine months (86.7%, 88.9%, and 89.5%, respectively) (Table 3). Specifically, among physician TB-presumptive CXRs, the proportion of concordant CAD TB-presumptive CXRs increased from 47.3% to 69.1% and 76.1%. Concordance was high between physician and CAD non-TB CXR results throughout the nine months of implementation.

### 3.5. Monitoring qXR Performance and Threshold Scores

The yield for Xpert-confirmed TB disease increased from 858 to 2112 per 100,000 CXRs across nine months (Table 4). Pseudo-sensitivity for CAD performance at qXR = 0.60 was higher than pseudo-specificity for detecting TB-presumptive CXRs among people with Xpert-confirmed TB. Pseudo-accuracy decreased over the nine months of implementation. These terms are described as “pseudo” values due to the algorithmic 2X implementation for which CXRs with abnormality scores below the CAD TB threshold were not systematically referred for Xpert testing. Optimal thresholds constrained by >95% pseudo-sensitivity were slightly higher, and more specific, than the implementation CAD TB threshold (≥0.60).

## 4. Discussion

In Vietnam, CAD integration into TB case finding supported physicians in district facilities to improve the quality of CXR interpretation and increase the yield for TB detection. Concordance between CXR results from physician and CAD interpretation improved with training and was important to optimize decisions for Xpert testing.

### 4.1. Using a CAD Programmatic Framework to Guide Non-Research Implementation

An operational research toolkit is available to support the effective use of CAD for TB, guiding threshold score calibration using a bacteriological reference standard [8]. For non-research programmatic implementation, there is limited guidance on how to evaluate CAD performance when diagnostic testing is not systematic below the CAD TB threshold. Approaches that have been used to evaluate programmatic implementation include estimating TB detection rates when adding CAD as a second reader [34] and measuring associations between CXR abnormality scores and Xpert positivity when pre-screening with CAD in a high TB-burden setting [35].

Our framework for programmatic implementation guided the design of CAD integration into the TB workflow, the analytical approach, and interpretation of results. The first step of implementation was selecting the model to integrate CAD into TB case finding. The CAD-parallel model was a pragmatic choice for the facility setting; in reality, district-level physicians, who have less expertise than at provincial and national levels, would likely have benefited from the “CAD-first” model in which CAD interpretation is sequential before the physician interpretation, and only CXRs read as CAD TB-presumptive are interpreted again by physicians. The CAD-first model provides more quality assurance (while also being more dependent upon CAD threshold performance), but the facility setting was not conducive to this sequential approach due to the separation of decision-making at various points in the TB workflow. Our findings show that CAD clinical decision support depends upon human factors such as physician concordance, which improves with training and is affected by the order in which CAD is integrated into the clinical workflow. CAD guidance for CXR interpretation should account for human factors to optimize clinical decision support [36,37].

Programmatic CAD implementation can be evaluated by simple analyses to measure the rates of TB-presumptive CXRs by physician and CAD; rates of Xpert testing and Xpert positivity; and agreement between physician and CAD interpretation of CXRs. In Vietnam’s district facilities, physicians were less inclined than CAD to read CXRs as TB-presumptive, as seen by the physicians’ lower rates of TB-presumptive results. In addition, when the physician disagreed with the CAD TB-presumptive result, Xpert testing was not ordered. It was also not unusual for the physician referring for Xpert testing to be different than the physician reading the CXRs, further challenging the quality of decisions for Xpert referral. It is possible that some CAD TB-presumptive CXRs were misread by the CAD software (e.g., external foreign body over the lung fields identified as a TB-presumptive abnormality), and the physicians correctly excluded those results before Xpert testing. However, several findings support the overall accuracy of CAD TB-presumptive CXR results; namely, that increased agreement between physician and CAD TB-presumptive CXRs resulted in an increased rate of Xpert testing and increased yield for Xpert-confirmed TB. Our findings also show that CXRs read as TB-presumptive by physicians alone had very low Xpert positivity rates; this could be from incorrect CXR interpretation by the physician or inaccurate screening for TB symptoms and risks.

Evaluating Xpert testing rates for CXRs with CAD abnormality scores above and below the CAD TB threshold can also inform how physicians refer individuals for diagnostic testing. The rate of Xpert testing for CAD non-TB CXRs (which are likely unnecessary tests) decreased but was still nearly 30% after nine months. Equally concerning was the high rate of “missed Xpert testing” for CAD TB-presumptive CXRs, which was 72.5% at three months and 56.6% at nine months. TB diagnoses were likely missed among these individuals who did not receive Xpert testing but who had CXRs that were read as TB-presumptive by CAD. Overall, our findings suggest that the decision to obtain Xpert testing should be guided by CAD TB-presumptive CXRs and that a higher proportion of TB-vulnerable populations should be tested with Xpert for facility-based 2X case finding.

### 4.2. Monitoring the CAD Threshold Score and Evaluating CAD Performance

The CAD TB threshold score should be selected prior to implementation and then fine-tuned and optimized as needed, based on the results. The selected qXR threshold ≥ 0.60 was guided by community 2X campaigns that preceded this implementation study, and it was set higher than the manufacturer’s pre-set threshold in order to increase specificity given that both physicians and CAD interpreted all CXRs. Threshold monitoring using > 95% pseudo-sensitivity targeting 20% PPV showed that the optimal threshold (0.617–0.654) could be increased from the implementation threshold (0.60) for higher specificity without sacrificing sensitivity. It is important to note that for CAD-parallel implementation, the concordance between physician and CAD interpretations is just as important as threshold score calibration, since physicians interpreted all CXRs and had the autonomy to refer for Xpert testing based on their own results (with or without consideration of the CAD result). As such, on-site training for physicians to understand and use the CAD results should be carried out prior to calibrating the threshold, to account for human factors. Responses to optimal threshold monitoring could also comprise both CAD and non-CAD adjustments; for example, specificity could be increased by raising CAD thresholds, or by adding clinical evaluation (symptoms, history, risk factors) as an additional step to guide decisions for diagnostic testing. Ultimately, threshold score monitoring and calibration should be informed by goals for TB screening or triage and the availability of budget and supplies for confirmatory diagnosis.

CAD diagnostic performance is ideally evaluated against bacteriological reference standards [8]. When analyzing programmatic data in which Xpert testing is indicated by CAD TB-presumptive CXRs and not carried out for all CXRs (i.e., testing is not systematic for normal CXRs), PRC and ROC curves and their AUCs can only estimate performance; sensitivity is likely over-estimated for programmatic results. In general, the PRC is more accurate than ROC curves for imbalanced datasets in which one population (positive class) is much smaller than the other (negative class) [38]. However, ROC curves are not affected by disease prevalence in the tested population and utilize a fixed baseline of 0.50 for balanced distribution from which to calculate the AUC. In contrast, the PRC is affected by disease prevalence in the tested population, and its baseline is not fixed but is equal to the proportion belonging to the positive class [38], i.e., Xpert positivity. Thus, variable disease prevalence and positivity rates will result in variable PRAUCs, and high prevalence will “raise the bar” from which the PRAUC is calculated. The high and variable Xpert positivity rates in this implementation study may therefore render PRAUCs less optimal than AUROCs, notwithstanding the ROC limitations for unbalanced datasets.

PRC and ROC curves have known advantages and disadvantages for evaluating CAD performance; optimal thresholds selected from these curves traditionally reflect the best trade-off between sensitivity and specificity for ROC curves, or precision (PPV) and recall (sensitivity) for PRCs. Our approach for selecting the optimal threshold prioritized sensitivity and then used PPV to “fine-tune” the threshold selection, as long as the optimal threshold’s sensitivity was greater than 95%. Knowing that programmatic CAD’s pseudo-sensitivity will be higher than the true sensitivity, we selected 95% pseudo-sensitivity for the optimal threshold, aiming for performance within the range of 90% sensitivity, the recommended Target Product Profile. The Vietnam NTP prioritizes sensitivity above specificity, with the goal to detect everyone with TB disease, including those who may have early or asymptomatic TB.

qXR at a threshold score ≥ 0.60 had high pseudo-sensitivity and low pseudo-specificity for Xpert-confirmed TB in district facility settings in Vietnam. In other countries, studies in tertiary [39] and primary [40] health facilities have also shown high CAD sensitivity and low specificity. Of note, performance metrics may not be generalizable across settings depending upon the CAD software and version, and population characteristics such as HIV prevalence, history of prior TB, and age. There is increasing evidence that optimized threshold scores vary for different vulnerable populations and settings [41,42], but the implications for implementation are unclear. Using specific thresholds for each population may not be pragmatic at the field and facility level, depending on the CAD software, how easily thresholds are adjusted, and the required monitoring to ensure quality. Our framework is one approach to analyze and interpret programmatic data when using CAD in non-research implementation. Programmatic experience from other countries and settings will help to inform the best analytical approach for real-world CAD implementation.

### 4.3. Limitations

Our implementation study had limitations. We did not collect sputum for Xpert analysis in all participants, due to the programmatic design; the bacteriological reference was thus only available for individuals with TB-presumptive CXRs or TB symptoms. Physicians were not blinded to the CAD interpretation, and it was not possible to know if or when they considered the CAD result in their CXR interpretations. Individual-level demographic and clinical characteristics were not available for the TB-vulnerable populations in facility 2X implementation, limiting our ability to conduct descriptive and subgroup analyses. We used a single-spot sputum specimen for Xpert testing, not culture; both factors could lead to underestimating TB prevalence. Finally, a formal cost-effectiveness analysis of CAD implementation was not conducted as part of this study and is needed to guide scalability and sustainability for Vietnam.

## 5. Conclusions

Our programmatic framework effectively integrates CAD into the TB diagnostic workflow in district facilities in Vietnam. The framework supports physicians to improve the quality of CXR interpretation and referral decisions for Xpert testing. CAD clinical decision support depends upon human factors such as physician-CAD concordance, which improves with training.

## Figures and Tables

**Table 1:** CXR and Xpert results analyzed by physician and CAD interpretation.

	April–June 2022	July–September 2022	October– December 2022
Number of district facilities implementing CAD	5	7	8
Total number of people with CXRs (*N*)	5826	9696	9423
CAD TB-presumptive CXR result (*n* [%])	749 (12.9)	1363 (14.1)	1538 (16.3)
Physician TB-presumptive CXR result (*n* [%])	480 (8.2)	755 (7.8)	1046 (11.1)
Difference of percentages (*p*-value) *	4.6 (<0.001)	6.3 (<0.001)	5.2 (<0.001)
Total number of people with valid Xpert tests (*N*)	455	703	903
Xpert positivity rate overall (*n* positive/*N* valid Xpert tests [%])	50/455 (11.0)	148/703 (21.1)	199/903 (22.0)
Xpert positivity rate for CAD and physician TB-presumptive CXRs (*n* positive/*n* Xperts with CAD and physician TB-presumptive CXRs [%])	48/206 (23.3)	143/472 (30.3)	196/659 (29.7)
Xpert positivity rate for CAD non-TB and physician TB-presumptive CXRs (*n* positive/*n* Xperts with physician TB-presumptive CXRs [%])	2/249 (0.8)	5/229 (2.2)	3/242 (1.2)

* Test of proportions for CAD TB-presumptive CXRs vs. physician TB-presumptive CXRs. qXR TB threshold ≥ 0.60. Trace positive Xperts were not counted as valid Xpert tests.

**Table 2:** Analysis of Xpert testing rates by CXR scores relative to the CAD TB threshold.

	April–June 2022	July–September 2022	October– December 2022
Total number of people with valid Xpert tests (*N*)	455	703	903
Xpert testing rate (*N* valid Xpert tests/*N* total CXRs [%])	455/5826 (7.8)	703/9696 (7.3)	903/9423 (9.6)
*n* Xpert tests not done for CXR abnormality score ≥ 0.60/*n* CXRs with score ≥ 0.60 (%)	543/749 (72.5)	887/1359 (65.3) *	830/1466 (56.6) *
*n* Xpert tests done for CXR abnormality score < 0.60/*N* valid Xpert tests (%)	249/455 (54.7)	231/703 (32.9) *	267/903 (29.6) *

* Test of proportions for April–June 2022 vs. July–September 2022 and October–December 2022, *p* < 0.001. qXR TB threshold ≥ 0.60. Trace positive Xpert results were not counted as valid Xpert tests.

**Table 3:** Agreement between physician and CAD CXR results.

	April–June 2022	July–September 2022	October– December 2022
Physician TB-presumptive CXR and CAD non-TB CXR (*n* [%])	253 (52.7)	233 (30.9) *	250 (23.9) *
Physician and CAD TB-presumptive CXR (*n* [%])	227 (47.3)	522 (69.1) *	796 (76.1) *
Physician non-TB CXR and CAD TB-presumptive CXR (*n* [%])	522 (9.8)	841 (9.4)	742 (8.9)
Physician non-TB CXR and CAD non-TB CXR (*n* [%])	4824 (90.2)	8100 (90.6)	7635 (91.1)
Total agreement (%), (Kappa [Standard error])	86.7% (0.30 [0.01])	88.9% * (0.44 [0.01])	89.5% * (0.56 [0.01])

* Test of proportions for April–June 2022 vs. July–September 2022 and October–December 2022, *p* < 0.001. qXR TB threshold ≥ 0.60. Trace positive Xpert results were not counted as valid Xpert tests.

**Table 4:** Evaluation of qXR performance for 2X facility case finding.

	April–June 2022	July–September 2022	October–December 2022
Total number of people with CXRs	5826	9696	9423
Number with CXR and Xpert results	455	703	903
Number with Xpert-positive results	50	148	199
Yield for Xpert-confirmed TB/100,000 CXRs	858	1526	2112
Pseudo-sensitivity at qXR = 0.60 (95% CI)	96.0% (90.0–100)	96.6% (93.9–99.3)	98.5% (96.5–100)
Pseudo-specificity at qXR = 0.60 (95% CI)	61.0% (56.3–65.9)	40.7% (36.6–45.1)	37.5% (34.0–41.1)
PPV at qXR = 0.60 (95% CI)	23.4% (21.1–25.9)	30.3% (28.8–32.1)	30.8% (29.6–32.1)
Pseudo-accuracy at qXR = 0.6 (95% CI)	64.8% (60.7–69.2)	52.5% (49.2–56.1)	50.9% (48.2–53.7)
AUROC (95% CI)	0.8598 (0.8127–0.9068)	0.8267 (0.7920–0.8614)	0.8062 (0.7755–0.8368)
PRAUC	0.4375	0.5755	0.4770
Optimal qXR threshold score at >95% pseudo-sensitivity	0.615	0.677	0.654

All analyses used Xpert as the bacteriological reference standard. Confidence intervals were not calculated for PRAUC. Trace positive Xpert results were excluded. AUROC = area under the receiver operating characteristics curve; CI = confidence interval; PPV = positive predictive value; PRAUC = precision recall area under the curve.

## Data Availability

The data that support the findings of this study are available from the Vietnam National TB Program; restrictions apply to the availability of these data, which were used with permission for the current study and are not publicly available. Data are available upon reasonable request to the corresponding author and with permission from the National TB Program.

## Supplementary Materials

The computer code that was developed to generate all results for this manuscript can be downloaded at: https://github.com/anhinnes/CAD-TB-Vietnam-2023.

## References

[1] World Health Organization (WHO) (2022). Global Tuberculosis Report 2022.

[2] Miller C., Lonnroth K., Sotgiu G., Migliori G.B. (2017). The long and winding road of chest radiography for tuberculosis detection. Eur. Respir. J..

[3] Harris M., Qi A., Jeagal L., Torabi N., Menzies D., Korobitsyn A., Pai M., Nathavitharana R.R., Ahmad Khan F. (2019). A systematic review of the diagnostic accuracy of artificial intelligence-based computer programs to analyze chest x-rays for pulmonary tuberculosis. PLoS ONE.

[4] Singh R., Kalra M.K., Nitiwarangkul C., Patti J.A., Homayounieh F., Padole A., Rao P., Putha P., Muse V.V., Sharma A. (2018). Deep learning in chest radiography: Detection of findings and presence of change. PLoS ONE.

[5] Murphy K., Habib S.S., Zaidi S.M.A., Khowaja S., Khan A., Melendez J., Scholten E.T., Amad F., Schalekamp S., Verhagen M. (2020). Computer aided detection of tuberculosis on chest radiographs: An evaluation of the CAD4TB v6 system. Sci. Rep..

[6] Qin Z.Z., Ahmed S., Sarker M.S., Paul K., Adel A.S.S., Naheyan T., Barrett R., Banu S., Creswell J. (2021). Tuberculosis detection from chest x-rays for triaging in a high tuberculosis-burden setting: An evaluation of five artificial intelligence algorithms. Lancet Digit. Health.

[7] World Health Organization (WHO) (2021). WHO CONSOLIDATED GUIDELINES on Tuberculosis. Module 2: Screening-Systematic Screening for Tuberculosis Disease.

[8] World Health Organization (WHO) (2021). Determining the Local Calibration of Computer-Assisted Detection (CAD) Thresholds and Other Parameters: A Toolkit to Support the Effective Use of CAD for TB Screening.

[9] Fanni S.C., Marcucci A., Volpi F., Valentino S., Neri E., Romei C. (2023). Artificial intelligence-based software with CE mark for chest X-ray interpretation: Opportunities and challenges. Diagnostics.

[10] Khan F.A., Majidulla A., Tavaziva G., Nazish A., Abidi S.K., Benedetti A., Menzies D., Johnston J.C., Khan A.J., Saeed S. (2020). Chest x-ray analysis with deep learning-based software as a triage test for pulmonary tuberculosis: A prospective study of diagnostic accuracy for culture-confirmed disease. Lancet Digit. Health.

[11] Qin Z.Z., Sander M.S., Rai B., Titahong C.N., Sudrungrot S., Laah S.N., Adhikari L.M., Carter E.J., Puri L., Codlin A.J. (2019). Using artificial intelligence to read chest radiographs for tuberculosis detection: A multi-site evaluation of the diagnostic accuracy of three deep learning systems. Sci. Rep..

[12] Fehr J., Gunda R., Siedner M.J., Hanekom W., Ndung U.T., Grant A., Lippert C., Wong E.B. (2023). CAD4TB software updates: Different triaging thresholds require caution by users and regulation by authorities. Int. J. Tuberc. Lung Dis..

[13] Qin Z.Z., Barrett R., Ahmed S., Sarker M.S., Paul K., Adel A.S.S., Banu S., Creswell J. (2022). Comparing different versions of computer-aided detection products when reading chest X-rays for tuberculosis. PLOS Digit. Health.

[14] World Health Organization (WHO) (2021). WHO operational handbook on tuberculosis. Module 2: Screening-Systematic Screening for Tuberculosis Disease.

[15] Recht M.P., Dewey M., Dreyer K., Langlotz C., Niessen W., Prainsack B., Smith J.J. (2020). Integrating artificial intelligence into the clinical practice of radiology: Challenges and recommendations. Eur. Radiol..

[16] Kapoor N., Lacson R., Khorasani R. (2020). Workflow applications of artificial intelligence in radiology and an overview of available tools. J. Am. Coll. Radiol..

[17] Duong M.T., Rauschecker A.M., Rudie J.D., Chen P.H., Cook T.S., Bryan R.N., Mohan S. (2019). Artificial intelligence for precision education in radiology. Br. J. Radiol..

[18] Hadley T.D., Pettit R.W., Malik T., Khoei A.A., Salihu H.M. (2020). Artificial intelligence in global health—A framework and strategy for adoption and sustainability. Int. J. MCH AIDS.

[19] Bizzo B.C., Almeida R.R., Michalski M.H., Alkasab T.K. (2019). Artificial Intelligence and Clinical Decision Support for Radiologists and Referring Providers. J. Am. Coll. Radiol..

[20] Lee H.W., Jin K.N., Oh S., Kang S.Y., Lee S.M., Jeong I.B., Son J.W., Han J.H., Heo E.Y., Lee J.G. (2023). Artificial Intelligence Solution for Chest Radiographs in Respiratory Outpatient Clinics: Multicenter Prospective Randomized Clinical Trial. Ann. Am. Thorac. Soc..

[21] World Health Organization (WHO) Tuberculosis Profile: Viet Nam. https://worldhealthorg.shinyapps.io/tb_profiles/?_inputs_&entity_type=%22country%22&lan=%22EN%22&iso2=%22VN%22.

[22] Steingart K.R., Schiller I., Horne D.J., Pai M., Boehme C.C., Dendukuri N. (2014). Xpert^®^ MTB/RIF assay for pulmonary tuberculosis and rifampicin resistance in adults. Cochrane Database Syst. Rev..

[23] Nishikiori N., Van Weezenbeek C. (2013). Target prioritization and strategy selection for active case-finding of pulmonary tuberculosis: A tool to support country-level project planning. BMC Public Health.

[24] Mac T.H., Phan T.H., Nguyen V.V., Dong T.T.T., Le H.V., Nguyen Q.D., Nguyen T.D., Codlin A.J., Mai T.D.T., Forse R.J. (2020). Optimizing Active Tuberculosis Case Finding: Evaluating the Impact of Community Referral for Chest X-ray Screening and Xpert Testing on Case Notifications in Two Cities in Viet Nam. Trop. Med. Infect. Dis..

[25] Nguyen L.H., Codlin A.J., Vo L.N.Q., Dao T., Tran D., Forse R.J., Vu T.N., Le G.T., Luu T., Do G.C. (2020). An Evaluation of Programmatic Community-Based Chest X-ray Screening for Tuberculosis in Ho Chi Minh City, Vietnam. Trop. Med. Infect. Dis..

[26] Hoa N.B., Sy D.N., Nhung N.V., Tiemersma E.W., Borgdorff M.W., Cobelens F.G. (2010). National survey of tuberculosis prevalence in Viet Nam. Bull. World Health Organ..

[27] Nguyen H.V., Tiemersma E.W., Nguyen H.B., Cobelens F.G.J., Finlay A., Glaziou P., Dao C.H., Mirtskhulava V., Nguyen H.V., Pham H.T.T. (2020). The second national tuberculosis prevalence survey in Vietnam. PLoS ONE.

[28] The Stop TB Partnership, FIND Resource Centre on Computer-Aided Detection Products for the Diagnosis of Tuberculosis. https://www.ai4hlth.org/.

[29] StataCorp (2021). Stata Statistical Software: Release 17, 17.

[30] R Core Team (2023). R: A Language and Environment for Statistical Computing, 4.3.0.

[31] Robin X., Turck N., Hainard A., Tiberti N., Lisacek F., Sanchez J.C., Muller M. (2011). pROC: An open-source package for R and S+ to analyze and compare ROC curves. BMC Bioinform..

[32] Saito T., Rehmsmeier M. (2017). Precrec: Fast and accurate precision-recall and ROC curve calculations in R. Bioinformatics.

[33] The Stop TB Partnership (2021). Screening and Triage for TB Using Computer-Aided Detection (CAD) Technology and Ultra-Portable X-ray Systems: A Practical Guide.

[34] Philipsen R., Sanchez C.I., Melendez J., Lew W.J., van Ginneken B. (2019). Automated chest X-ray reading for tuberculosis in the Philippines to improve case detection: A cohort study. Int. J. Tuberc. Lung Dis..

[35] Madhani F., Maniar R., Burfat A., Ahmed M., Farooq S., Sabir A., Domki A., Page-Shipp L., Khowaja S., Safdar N. (2020). Automated chest radiography and mass systematic screening for tuberculosis. Int. J. Tuberc. Lung Dis..

[36] Knop M., Weber S., Mueller M., Niehaves B. (2022). Human Factors and Technological Characteristics Influencing the Interaction of Medical Professionals With Artificial Intelligence-Enabled Clinical Decision Support Systems: Literature Review. JMIR Hum. Factors.

[37] U.S. Food & Drug Administration, Health Canada, Medicines & Healthcare Products Regulatory Agency (2021). Good Machine Learning Practice for Medical Device Development: Guiding Principles.

[38] Saito T., Rehmsmeier M. (2015). The precision-recall plot is more informative than the ROC plot when evaluating binary classifiers on imbalanced datasets. PLoS ONE.

[39] Nash M., Kadavigere R., Andrade J., Sukumar C.A., Chawla K., Shenoy V.P., Pande T., Huddart S., Pai M., Saravu K. (2020). Deep learning, computer-aided radiography reading for tuberculosis: A diagnostic accuracy study from a tertiary hospital in India. Sci. Rep..

[40] Muyoyeta M., Maduskar P., Moyo M., Kasese N., Milimo D., Spooner R., Kapata N., Hogeweg L., van Ginneken B., Ayles H. (2014). The sensitivity and specificity of using a computer aided diagnosis program for automatically scoring chest X-rays of presumptive TB patients compared with Xpert MTB/RIF in Lusaka Zambia. PLoS ONE.

[41] Geric C., Qin Z.Z., Denkinger C.M., Kik S.V., Marais B., Anjos A., David P.M., Ahmad Khan F., Trajman A. (2023). The rise of artificial intelligence reading of chest X-rays for enhanced TB diagnosis and elimination. Int. J. Tuberc. Lung Dis..

[42] Tavaziva G., Harris M., Abidi S.K., Geric C., Breuninger M., Dheda K., Esmail A., Muyoyeta M., Reither K., Majidulla A. (2022). Chest X-ray analysis with deep learning-based software as a triage test for pulmonary tuberculosis: An individual patient data meta-analysis of diagnostic accuracy. Clin. Infect. Dis..

